# Assessment of Centre National d’Études Spatiales Real-Time Ionosphere Maps in Instantaneous Precise Real-Time Kinematic Positioning over Medium and Long Baselines

**DOI:** 10.3390/s20082293

**Published:** 2020-04-17

**Authors:** Dariusz Tomaszewski, Paweł Wielgosz, Jacek Rapiński, Anna Krypiak-Gregorczyk, Rafał Kaźmierczak, Manuel Hernández-Pajares, Heng Yang, Raul OrúsPérez

**Affiliations:** 1 Faculty of Geoengineering, University of Warmia and Mazury in Olsztyn, Oczapowskiego str. 2, 10-719 Olsztyn, Poland; pawel.wielgosz@uwm.edu.pl (P.W.); jacek.rapinski@uwm.edu.pl (J.R.); a.krypiak-gregorczyk@uwm.edu.pl (A.K.-G.); rafal.kazmierczak@uwm.edu.pl (R.K.); 2 Department of Mathematics, UPC-IonSAT & IEEC-UPC, Universitat Politècnica de Catalunya, 08034 Barcelona, Spain; manuel.hernandez@upc.edu (M.H.-P.); h.yang@upc.edu (H.Y.); 3 ESTEC, European Space Agency, 2200 AG Noordwijk, The Netherlands; raul.orus.perez@esa.int

**Keywords:** GNSS, ionosphere, RTK, SSR

## Abstract

Precise real-time kinematic (RTK) Global Navigation Satellite System (GNSS) positioning requires fixing integer ambiguities after a short initialization time. Originally, it was assumed that it was only possible at a relatively short distance from a reference station (<10 km), because otherwise the atmospheric effects prevent effective ambiguity fixing. Nowadays, through the use of VRS, MAC, or FKP corrections, the distances to the closest reference station have been increased to around 35 km. However, the baselines resolved in real time are not as far as in the case of static positioning. Further extension of the baseline requires the use of an ionosphere-weighted model with ionospheric delay corrections available in real time. This solution is now possible thanks to the Radio Technical Commission for Maritime (RTCM) stream of SSR corrections from, for example, Centre National d’Études Spatiales (CNES), the first analysis center to provide it in the context of the International GNSS Service. Then, ionospheric delays are treated as pseudo-observations that have a priori values from the CLK RTCM stream. Additionally, satellite orbit and clock errors are properly considered using space-state representation (SSR) real-time radial, along-track, and cross-track corrections. The following paper presents the initial results of such RTK positioning. Measurements were performed in various field conditions reflecting realistic scenarios that could have been experienced by actual RTK users. We have shown that the assumed methodology was suitable for single-epoch RTK positioning with up to 82 km baseline in solar minimum (30 March 2019) mid and high latitude (Olsztyn, Poland) conditions. We also confirmed that it is possible to obtain a rover position at the level of a few centimeters of precision. Finally, the possibility of using other newer experimental IGS RT Global Ionospheric Maps (GIMs), from Chinese Academy of Sciences (CAS) and Universitat Politècnica de Catalunya (UPC) among CNES, is discussed in terms of their recent performance in the ionospheric delay domain.

## 1. Introduction

Currently, the Global Navigation Satellite System (GNSS) real-time kinematic (RTK) is one of the most popular positioning methods in geodesy and surveying. The widespread use of RTK is because it is a method that obtains accuracy at the level of a few centimeters in real time (RT). The premise of RTK is the use of measurements from a reference station to determine the precise position of a “rover” receiver in a relative mode [1]. Commercial and national reference station networks have been established in many countries for, among other purposes, the effective use of the RTK method. Originally, using a single reference station, the distance from the receiver could not exceed 10 km [2]. Contemporary networks through the use of virtual reference station (VRS), master-auxiliary concept (MAC), or Flächen Korrectur parameter (FKP) solutions, operate at distances up to 35 km from the reference station [3,4,5,6]. Further research has shown that in order to extend the distance between the receiver and the reference station, it is necessary to change the positioning model and minimize errors affecting the accuracy of this positioning [7,8]. Most of these studies assumed the use of the ionosphere-weighted model [9,10] which allowed longer baselines by applying suitably balanced double-differentiated ionospheric correction (among others) in the positioning model [1,7,11]. These studies showed that it was possible to extend a baseline up to 100 km and, at the same time, obtain correct ambiguity resolutions. It should be noted, however, that the proposed solutions were usually presented as rapid-static, fast-static, kinematic, or even instantaneous (single-epoch) results calculated on the basis of post-processed data, mainly because they required ionospheric corrections obtained from the data available in IONEX files that are typically available in post-mission time. Nowadays, however, it is possible to use real-time space-state representation (SSR) ionospheric corrections provided via the Radio Technical Commission for Maritime (RTCM). These corrections are provided, among others but firstly, by the Centre National d’Études Spatiales (CNES) in the form of spherical harmonic expansion (SHE) coefficients through RT streams. Research on real-time SSR correction streams is mainly performed in relation to real-time precise point positioning (RT-PPP) [12,13,14]. The use of real-time global ionospheric maps (RT-GIMs) or other predicted ionosphere maps (in IONEX format) is most often also presented in relation to the PPP technique [15,16]. Studies on the use of global ionospheric maps in RTK positioning present very promising results, however, they are predominantly developed in postprocessing mode [17]. Therefore, the aim of the presented research was to test the applicability of the RTCM CLK90 (currently named SSRC00CNE0) in long-range instantaneous (singe-epoch) RTK positioning. In order to obtain representative results, the GPS observations were carried out in various measurement conditions, and the positioning was performed in actual real time. Furthermore, in Section 4 we present prospects of potential improvements in RT-GIM accuracy that would further benefit their application in precise positioning.

## 2. Materials and Methods

This study focused on the application of real-time SSR ionospheric corrections provided via RTCM streams to obtain single-epoch RTK positions at a distance of about 82 km from the reference station. The research was intended to demonstrate the applicability of RTCM CLK90 stream to medium-range instantaneous RTK positioning. Such a solution was possible thanks to the ionosphere-weighted positioning model [9,18,19] along with RTCM CLK90 CNES stream [20,21,22].

The processing of GPS measurements was carried out in two scenarios, i.e., in actual real time and in the postprocessing mode. The latter solution served as a reference. In the first scenario, the measurements from the RTCM 3.X streams were developed in real time using our pyGNSS software. This software makes it is possible to receive ionospheric corrections in real time using SSR vertical total electron content (VTEC) RTCM message (no. 1264), provided, for example, in CLK90 stream. In the second scenario, GINPOS postprocessing software was used [11,22,23]. This software has often been used to perform precise satellite positioning and it is a proven research tool. In general, GINPOS implements the same positioning algorithms as pyGNSS, however, it performs calculations in postprocessing mode. For this scenario, observational data were uploaded in RINEX files, and ionospheric corrections were calculated on the basis of IONEX files provided by the Universitat Politècnica de Catalunya (UPC) [24,25]. The GINPOS software was used to assess the results that were obtained in real time with pyGNSS.

The pyGNSS software was created by the Department of Geodesy at the University of the Warmia and Mazury in Olsztyn. This research software was developed entirely in Python 2.7. Its main task was to perform satellite positioning in real time using RTCM 3.X streams. Single point positioning (SPP), differential GPS (DGPS), and long-range instantaneous RTK positioning algorithms were implemented. The software was also able to calculate longer observation sessions in real time. Using the application was possible through Graphical User Interface (GUI) designed in QT4 through pyQT4 bindings. Access to the intermediate and final results was possible almost at any stage of processing.

**Figure 1:**
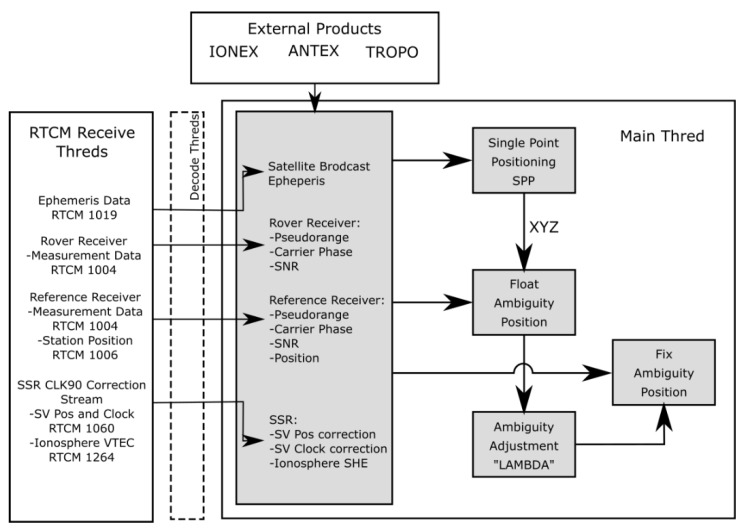
The data flow diagram of the pyGNSS software.

The pyGNSS software made it possible to use real-time SSR corrections, as well as data models stored, for example, in ANTEX and IONEX formats for phase center and ionospheric corrections, to augment performed estimations. The software operated according to the following scheme shown in Figure 1.

The purpose of the study was to process real-time kinematic medium-length baselines with 1-second measurement intervals using single-epoch GPS measurements. This approach is often called instantaneous positioning, as each epoch of data is being processed. For this reason, the ionosphere-weighted algorithm that uses double-differenced (DD) ionospheric delays as pseudo-observations in the positioning model, was implemented. The functional model also used double-differenced phase and code observables. The model is given as [26]:

$$\lambda_{1}\varphi_{1,ij}^{kl}-\rho_{ij}^{kl}-\left(\alpha_{i}^{k}ZTD_{i}-\alpha_{i}^{l}ZTD_{i}-\alpha_{j}^{k}ZTD_{j}+\alpha_{j}^{l}ZTD_{j}\;\right)+I_{ij}^{kl}-\lambda_{1}N_{1,ij}^{kl}=0\;\lambda_{2}\varphi_{2,ij}^{kl}-\rho_{ij}^{kl}-\left(\alpha_{i}^{k}ZTD_{i}-\alpha_{i}^{l}ZTD_{i}-\alpha_{j}^{k}ZTD_{j}+\alpha_{j}^{l}ZTD_{j}\;\right)+(f_{1}^{2}/f_{2}^{2})I_{ij}^{kl}-\lambda_{2}N_{2,ij}^{kl}=0\;P_{1,ij}^{kl}-\rho_{ij}^{kl}-\left(\alpha_{i}^{k}ZTD_{i}-\alpha_{i}^{l}ZTD_{i}-\alpha_{j}^{k}ZTD_{j}+\alpha_{j}^{l}ZTD_{j}\;\right)-I_{ij}^{kl}=0$$

where *i* and *j* are the receiver indexes; *k* and *l* are the satellite indexes; $\rho_{ij}^{kl}$ is the DD geometric range;$\;\varphi_{n,ij}^{kl}$ is the DD carrier-phase observable on frequency n;$\;P_{1,ij}^{kl}$ is the DD pseudo-range observable;$\;I_{ij}^{kl}$ is the DD ionospheric delay;$\;ZTD_{i}$ is the Zenith tropospheric delay;$\;\alpha_{i}^{}$ is the troposphere mapping function; $N_{n,ij}^{kl}$ is the carrier-phase ambiguities on frequency n; $f_{1}^{}\;\mathrm{and}\;f_{2}^{}$ are the GPS frequencies of the L1 and L2 signals; $\lambda_{1}\;\mathrm{and}\;\;\lambda_{2}$ are wavelengths of the L1 and L2 signals.

The model presented in the software requires dual-frequency carrier phase, and single-frequency pseudorange GPS observations. The unknown parameters are as follows:

- User receiver coordinates;
- Double-differenced (DD) ionospheric delays;
- Zenith tropospheric delays;
- Double-differenced (DD) integer ambiguities.

All the parameters are constrained to some a priori information, which can consist of empirical values. In the case of ionospheric corrections, a priori values were calculated on the basis of SHE coefficients provided in RTCM 3.X message 1264. For the calculation of tropospheric correction, the UNB3m model with Neil mapping function was selected to determine slant tropospheric corrections that were fixed in the processing [27,28,29].

To determine integer ambiguity values, a classic three-step solution was used as follows: A float solution with the ambiguities as real numbers in step one, an integer ambiguity search in second step, and a fixed solution in which real-valued ambiguities are replaced by the integers in step three [30,31]. The least-squares ambiguity decorrelation adjustment (LAMBDA) was used to fix the ambiguities to their integer values [32]. 

The most innovative feature introduced by the pyGNSS software is the use of SSR VTEC RTCM message (no. 1264) to obtain the ionospheric corrections in real time. VTEC, in this message, was provided using the SHE coefficients. The implementation was based on the following proposal of new RTCM SSR messages SSR Stage 2: VTEC for RTCM STANDARD 10403.2, differential GNSS, and services version 3 developed by RTCM special committee no. 104 [33]. According to this standard, the VTEC contribution is computed in TECU as:

$$VTEC\left(\phi_{PP},\lambda_{PP}\right)=\overset{N}{\underset{n=0}{\sum }}\overset{\min\left(n,M\right)}{\underset{m=0}{\sum }}\left(c_{nm}\cos\left(m\lambda_{s}\right)+s_{nm}\sin\left(m\lambda_{S}\right)\right)P_{nm}\left(sin\phi_{PP}\right)$$

where N is the degree of spherical expansion (DF474), M is the order of spherical expansion (DF475), n and m are indexes, c_nm_ is the cosine coefficient for the layer (DF476), s_nm_ is the sine coefficient for the layer (DF477), λ_s_ is the mean sun fixed and phase shifted longitude of ionospheric pierce point for the layer, λ_PP_ is the longitude of ionospheric pierce point for the layer, t is GPS time, φ_PP_ is geocentric latitude of ionospheric pierce point for the layer, and P_nm_( ) are the fully normalized associated Legendre functions.

## 3. Results

### 3.1. Experiment Description

The experiment was carried out on 30 April 2019 (DOY 120) between 9:30 am. and 10:30 am. During the test, a car was equipped with a GPS receiver, Internet communication, and a computer to record and process observational data. A Topcon NET-G5 receiver was used for this research. It is a high-class receiver primarily used for reference stations, so it was possible to save raw observation data in RINEX format, and also transfer RTCM streams using the NTRIP caster protocol at the same time. A Trimble zephyr 3 antenna was connected to the receiver. The Topcon receiver collected observations at 1 Hz frequency, data were recorded in the RINEX format, and in parallel transmitted as RTCM streams. The obtained GPS data were calculated in two scenarios:

- Real-time instantaneous RTK positioning based on RTCM streams using the pyGNSS software;
- Postprocessed instantaneous RTK using RINEX and IONEX files and calculated with the GINPOS software [23,24].

For the case of actual real-time positioning, during the tests, the receiver sent raw observation data (RTCM 1004) via the wireless Internet network to the computer running pyGNSS. At the same time, the computer received reference station data and the necessary SSR correction data from the remaining sources (Table 1). Note that we did not use any correction data from ground-based augmentation systems, for example, EUPOS [34,35].

**Figure 2:**
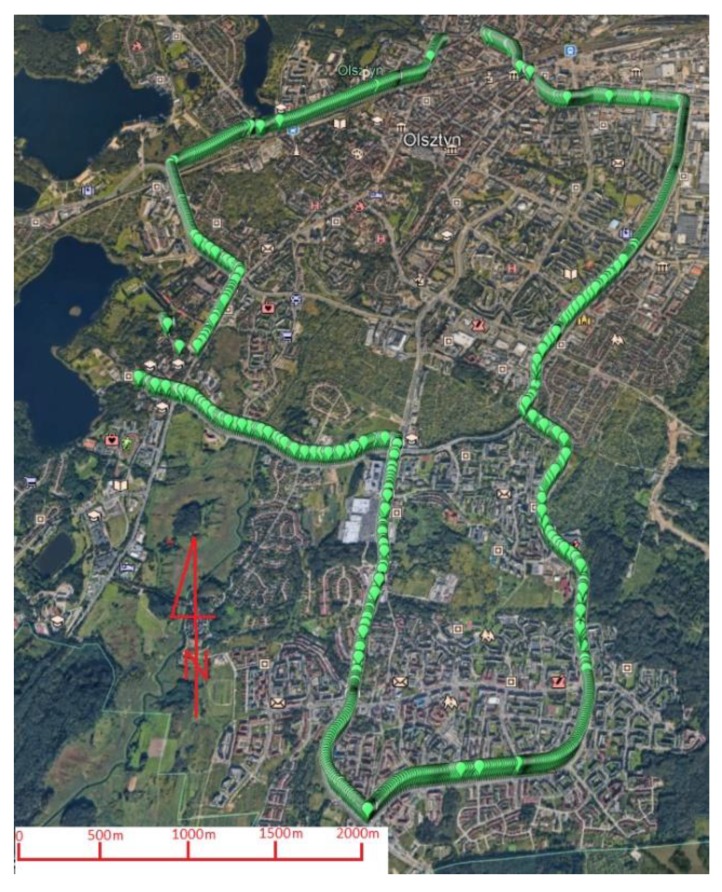
Google Earth view of the car test route (Olsztyn, Poland).

**Figure 3:**
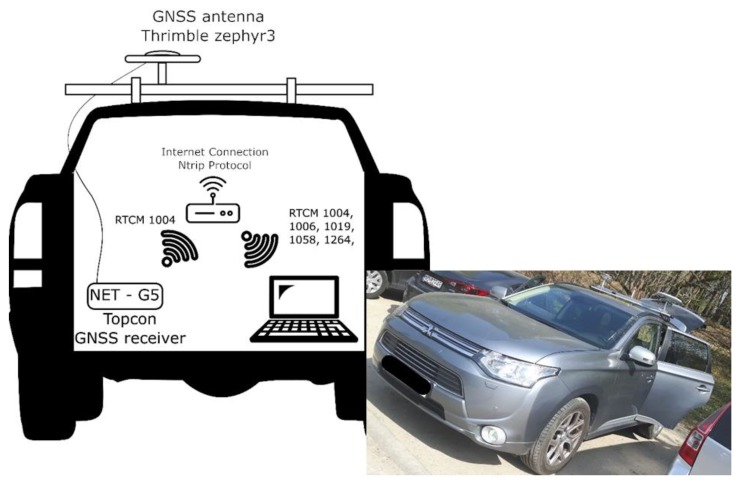
Car setup configuration during the test drive.

A car covered the route which was characterized by a variable exposure of the horizon. The test drive lasted about 50 minutes, which provided the opportunity to collect 3300 measuring epochs. Figure 2 presents a Google Earth view of the entire route. The route was selected so that the car experienced variable conditions. In the western and eastern parts, the car was driven through areas with a clear horizon, whereas, in the south, the area was characterized by a higher urban development, and in the north by two-to three-storey buildings. The influence of covering the horizon by the buildings is visible later on in the section describing the research results. The setup configuration of the car is presented in Figure 3.

### 3.2. PyGNSS Kinematic Real-Time Data Processing

During the tests, the RTCM data were streamed from the TOPCON receiver and the reference stations BROD and KRO1 were used (Table 1). Thus, short (<10 km) and long (~82 km) baselines were established for RTK positioning with SSR ionospheric corrections. The positioning solution was provided using the ionosphere-weighted relative positioning model and single-epoch (instantaneous) approach. This choice was due to the relatively low level of the ionospheric TEC, in the solar minimum and mid and high latitude ionospheric conditions, which made it relatively easy to solve the position instantaneously. However, the instantaneous solution was more sensitive to the quality of ionospheric corrections. It is notable that in this approach, each epoch is processed independently, i.e., no previous epoch information is used in the currently processed epoch. Finally, real-time data was processed in the following three variants:

- short baseline to KRO1 (1 to 6 km) that served as a reference solution;
- ~81.7 km baseline to the BROD reference station, without SSR ionospheric corrections;
- ~81.7 km baseline to the BROD reference station, with SSR ionospheric corrections (from 1264 RTCM message).

The same processing parameters were used in the case of both baselines (Table 2).

**Figure 4:**
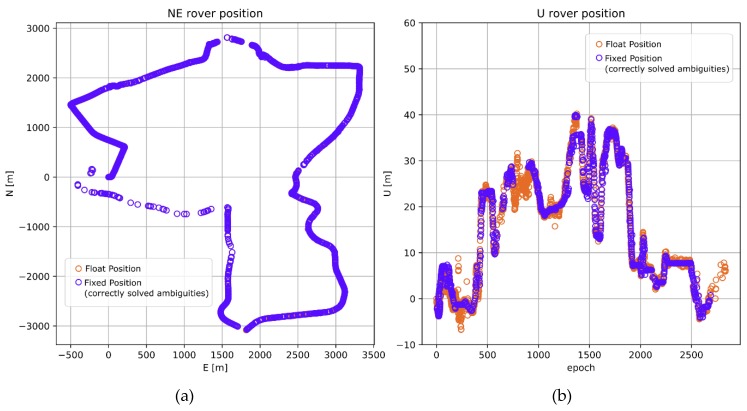
Reference rover trajectory derived from processing short baseline (KRO1). (**a**) Horizontal components; (**b**) Vertical components.

For comparative purposes, a reference trajectory was processed. Therefore, test kinematic data and observations from the closest reference station KRO1 (1 to 6 km) were evaluated. The reference solution obtained 86% correctly solved epochs, which resulted in a total of 2838 reference positions (Figure 4a,b). The ratio-test [36,37,38] was used to validate the results with a threshold of > 2.5. The resulting rover coordinates were transformed into topocentric coordinate frame (NEU components) and also served as reference results for long baseline processing (BROD). The accuracy of the reference trajectory was defined as 0.010 m for horizontal components and 0.015 m for the height component. This accuracy level was determined by comparing results of a one-hour session of static positioning to instantaneous RTK positioning performed with the same static data.

**Figure 5:**
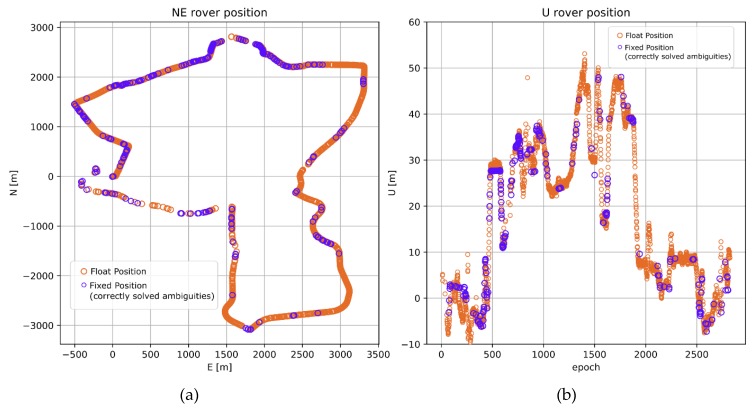
Rover trajectory from processing 82 km baseline (BROD), no ionospheric corrections. (**a**) Horizontal components; (**b**) Vertical components.

**Figure 6:**
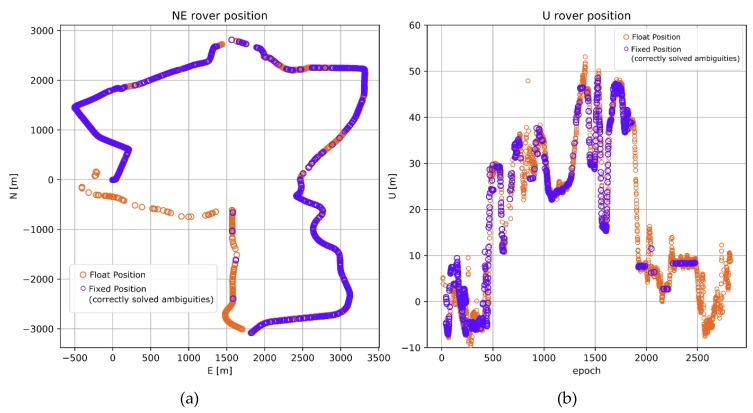
Rover trajectory from processing 82 km baseline (BROD), with Centre National d’Études Spatiales (CNES) real-time (RT) ionospheric corrections. (**a**) Horizontal components; (**b**) Vertical components.

With the use of the aforementioned processing parameters, the BORD baseline of almost 82 km was evaluated. Calculations were made in two variants. In the first one, no ionospheric corrections from RTCM message no. 1264 were used. In this variant, on the one hand, a priori DD ionospheric delays were set to zero in our ionosphere-weighted positioning model. The resulting trajectory is presented in Figure 5. In the second variant, on the other hand, the DD ionospheric correction values calculated from CLK90 CNES stream were used as a priori ionospheric delays in the positioning model. The resulting trajectory is shown in Figure 6.

**Figure 7:**
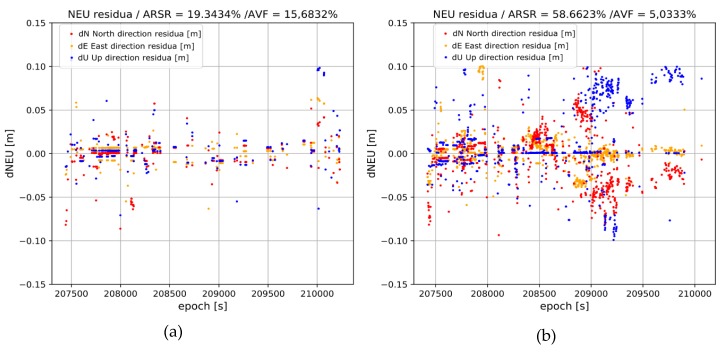
Rover position residua vs. the reference solution from processing the BROD baseline. (**a**) Without ionospheric corrections; (**b**) With CNES RT ionospheric corrections. Horizontal residua dE; dN are shown in orange and red, respectively; and blue depicts height component.

For both variants, the residuals relative to the reference trajectory were calculated in ENU (East-North-Up) reference frame (Figure 7). The residuals were computed only for “fixed” epochs, i.e., with correctly solved carrier phase ambiguities. 

Table 3 presents the statistics of the obtained results. The metrics used for statistical analysis of the positioning results are ambiguity resolution success rate (ARSR) and ambiguity validation failure ratio (AVRF). The ARSR presents the percentage of processed epochs with correctly fixed ambiguities, while the AVRF is the percentage of epochs with the ratio-test higher than that of the threshold (ratio-test > 2.5), but they were not correct (wrong ambiguity fixes). This was validated by the comparisons with the reference solution from the processing of the short baseline. Therefore, the ARSR_true parameter shows the externally validated success of the ambiguity resolution. 

On the one hand, the trajectory obtained by processing the BROD baseline without the use of ionospheric corrections is characterized by a very low ARSR_true (~17%) and high number of wrong fixes (~16%). Only 538 epochs from the collected measurement set were solved correctly. On the other hand, the use of ionospheric corrections in the model significantly improved the obtained results. As can be observed in Table 3, the success rate of the ambiguity resolution increased from ~17% to ~56%, which was 1838 epochs, gaining a three-fold improvement in the success ratio. More importantly, the AVRF parameter was reduced from ~15% to ~5%.

At the same time, in Figure 7, it can be noticed that the accuracy of the results obtained in both variants is quite similar. Slightly higher residuals are observed for the second variant. Note, however, that the solution with the CNES ionospheric corrections resulted in solving three times more epochs, which mostly concerned cases with a lower number of satellites (higher PDOP). The average values of the residuals’ oscillate were approximately 0.005 m and the standard deviations did not exceed 0.025 m. On the basis of the obtained results, it can be stated that the application of the RT CNES corrections, provided via RTCM stream for processing long-range kinematic solution, and improved the success rate and reliability of positioning results while maintaining the same accuracy.

### 3.3. GINPOS: Kinematic Data Processed in Postprocessing

Additionally, independent tests were carried out in the postprocessing mode using UWM GINPOS software developed in Matlab [39,40,41]. These tests were performed in order to provide another reference for the results obtained in real time. In this scenario, three reference stations were selected which resulted in the formation of short (<10 km), medium (~60 km), and long (~82 km) baselines. The solution was provided using the ionosphere-weighted relative positioning model with a single-epoch (instantaneous) approach, as was in the case of real-time scenario. The kinematic data described in Section 3.1 were processed in several variants as follows:

- A short baseline (1 to 6 km) that served as a reference solution;
- a 62 km baseline to the DZIA reference station, with and without ionospheric corrections;
- an 81.7 km baseline to the BROD reference station, with and without ionospheric corrections;
- multi-baseline (61, 62, and 81.7 km to MRAG, DZIA, and BROD), with and without ionospheric corrections.

In the case of all baselines, the same processing parameters were used (Table 4). In the postprocessing tests, however, the ionospheric corrections were derived from UQRG GIMs computed by UPC-IonSAT [25,26,42]. 

**Figure 8:**
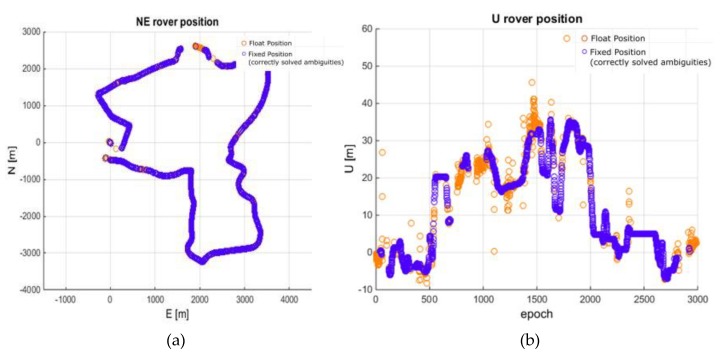
Reference rover trajectory derived from processing short baseline (KRO1). (**a**) Horizontal components; (**b**) Vertical components.

The reference solution was able to solve 77.5% of the epochs, i.e., with relatively conservative threshold of W-test > 3 (Figure 8) [43]. This threshold practically guaranteed 100% reliability of the ambiguity validation over baselines < 10 km [44]. The resulting rover coordinates were transformed to topocentric coordinate frame and served for the validation of the results from processing longer baselines.

**Figure 9:**
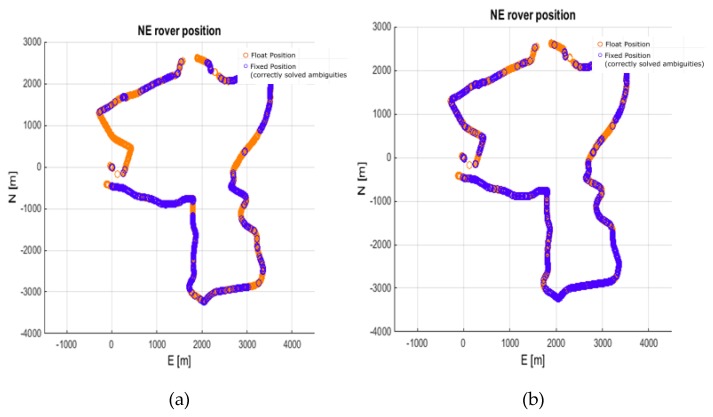
Rover trajectory from processing 62 km baseline (DZIA). (**a**) No ionospheric corrections; (**b**) With ionospheric corrections.

Subsequent determinations were made for the DZIA reference station. The DZIA solution was able to solve 35.6% of the epochs in the scenario without ionospheric correction, and 47.8% of the epochs in the variant with the ionospheric corrections. This study shows that for a 62 km baseline, 12.2% more epochs were correctly resolved with the use of ionospheric corrections. The results are shown in Figure 9. As for the reliability of the solution, in the first variant we obtained 0.87% wrong fixes and only 0.41% in the second variant. 

**Figure 10:**
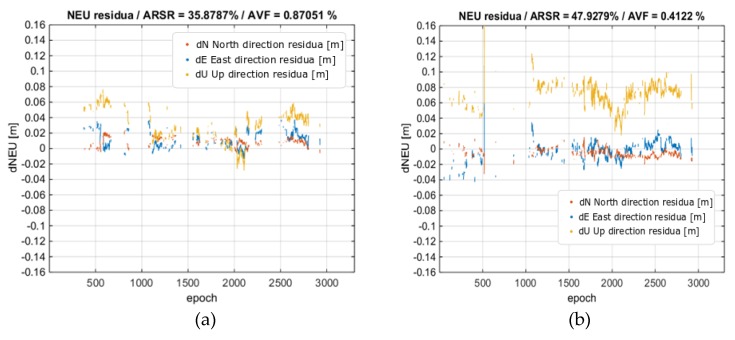
Rover position residua vs. the reference solution from processing DZIA baseline. (**a**) No ionospheric corrections; (**b**) With ionospheric corrections. Horizontal residuals are in red and blue, and height residuals are in yellow.

Figure 10 shows the residual values relative to a reference trajectory. As in the real-time scenario, the coordinates in the ENU coordinate frame were compared. 

**Figure 11:**
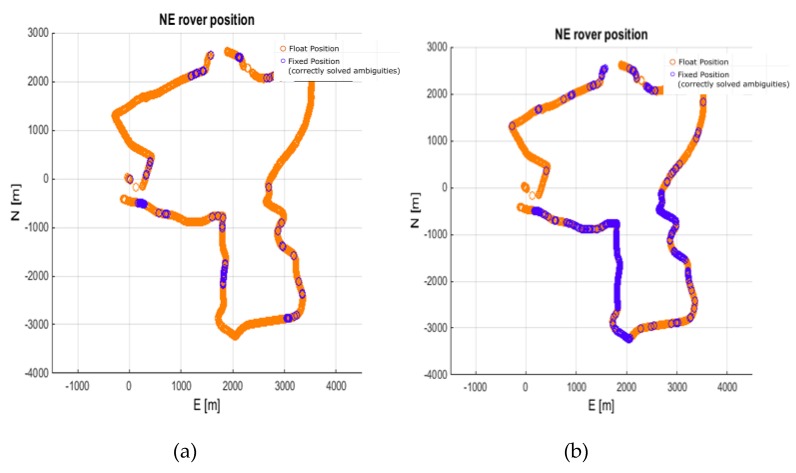
Rover trajectory from processing 82 km baseline (BROD). (**a**) No ionospheric corrections; (**b**) With ionospheric corrections.

**Figure 12:**
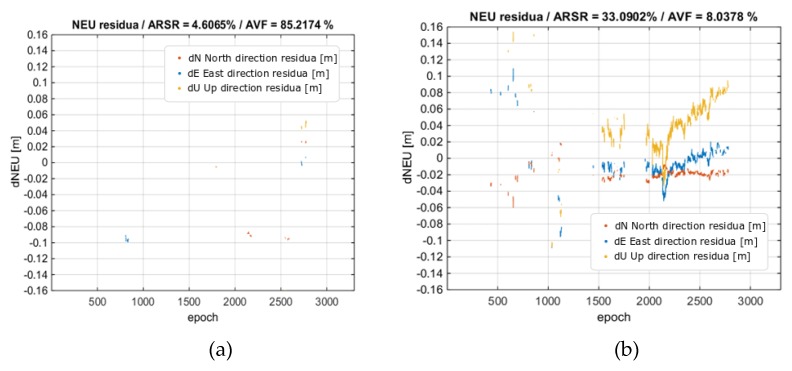
Rover position residua vs. the reference position from processing BROD baseline. (**a**) No ionospheric corrections; (**b**) With ionospheric corrections. Horizontal residuals are in red and blue, and height residuals are in yellow.

Another test was performed for the BROD reference station, similar to the case of the real-time solution with pyGNSS. Note, in this test, that the distance to the reference station was ~82 km. As in the previous tests, the calculations were made without and with ionospheric corrections. In the first variant, only 0.7% of the epochs were correctly fixed. After applying ionospheric corrections, the percentage of successes increased to 30.4%. However, a very clear influence of the ionospheric corrections was observed in the case of reliability of the solution. The percentage of wrong fixes dropped from 85% to 8% when the ionospheric corrections were applied. It is worth noting that the postprocessed solution is less effective as it was able to solve 30% of the epochs as compared with 55% in real time, due to a more conservative approach to the validation of the ambiguity selection. This difference could also come from some differences in the implementation of the ionosphere-weighted positioning and stochastic models. The trajectories of the obtained results are depicted in Figure 11. Figure 12 shows the residuals obtained in the BROD test variant. Similar to previous analyses, the data in the figure are provided for the fixed solutions only.

**Figure 13:**
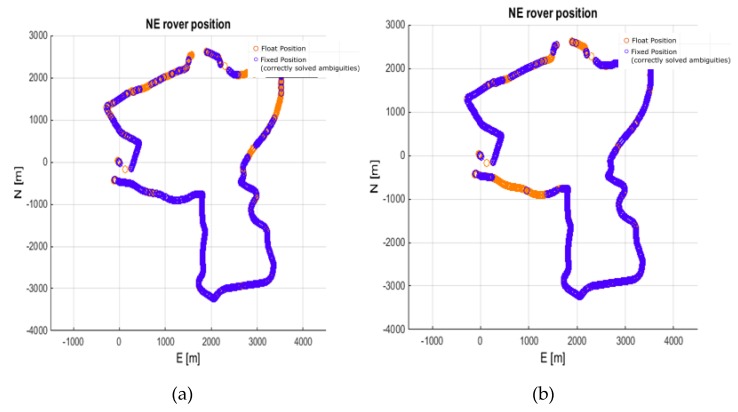
Rover trajectory from processing 62 to 82 km baselines (MULTI3). (**a**) No ionospheric corrections; (**b**) With ionospheric corrections.

**Figure 14:**
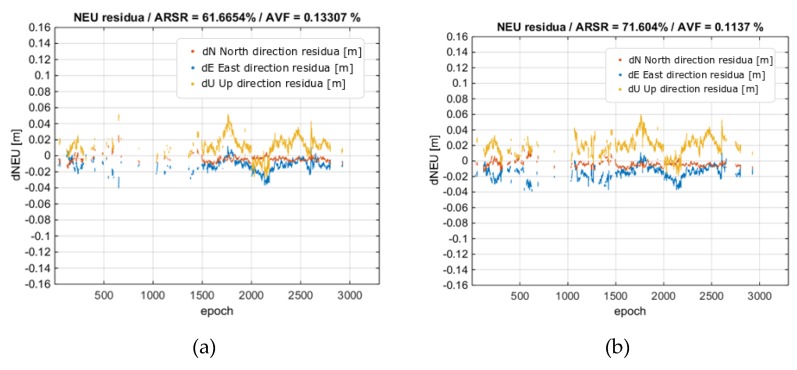
Rover position residua vs. the reference solution from processing MULTI3 solution. (**a**) No ionospheric corrections; (**b**) With ionospheric corrections. Horizontal residuals are in red and blue, and height residuals are in yellow.

The GINPOS software, however, was able to process a multi-baseline solution, i.e., when the data from several reference stations are adjusted in a single positioning model [11,27,45,46]. This strengthens the positioning model. Therefore, the last variant concerned the multi-baseline positioning using MRAG, DZIA, and BROD reference stations (denoted MULTI3). Their lengths ranged from 62 to 82 km. As expected, the results were clearly improved. The use of the multi-baseline solution resulted in a 62% success rate without ionospheric corrections and 72% with the application of corrections. More importantly, the reliability of the solution for both variants is high, i.e., the AVFR for the solution without and with ionospheric corrections dropped to 0.13% and 0.11%, respectively. The obtained trajectories are shown in Figure 13 and the residual analysis against reference solution is presented in Figure 14. Residua of the horizontal components of the fixed solution are within +/- 3 cm from the reference solution. In the case of the vertical component, the residuals are up to + 6 cm.

Table 5 presents the statistics of processed variants. The values used for statistical analysis are analogical to those in real-time tests. ARSR, ARSR_true, and AVRF were used.

## 4. Potential Improvements under IGS Experimental RT-GIMs

**Figure 15:**
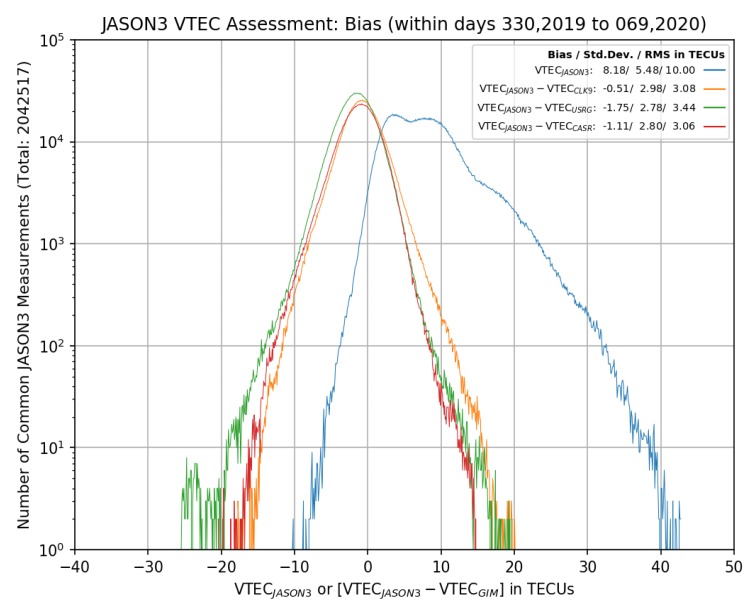
Histogram, in log scale for the number of counts, of the vertical total electron content (VTEC) difference of Jason–3 measurement minus real-time global ionospheric maps (RT-GIM) value during day 330 in 2019 to day 69 in 2020 for RT-GIMs of CNES (*CLK9*, orange), Universitat Politècnica de Catalunya (UPC)-IonSAT (*USRG*, green), and Chinese Academy of Sciences (CAS) (*CASR*, magenta),. The histogram of the reference values of Jason–3 is represented in blue. The corresponding bias, standard deviation, and RMS are indicated in the legend.

The results presented in Section 3 hold considerable promise for the application of RT-GIM in RTK positioning. However, there is an on-going effort by IGS to develop an official, operational RT product. The on-going development of three experimental IGS RT-GIMs, from CNES, CAS (Chinese Academy of Sciences, China), and UPC-IonSAT, and the corresponding experimental combination, is summarized in Section 4 of [47] from day 286 of 2018 to the 96th day of 2019, in terms of performance versus + 2,000,000 JASON3 altimeter VTEC measurements. Such results, showing an improvement of the RT-GIMs performance, is confirmed in our recent assessment of the three experimental IGS RT-GIMs from day 330 of 2019 to the 69th day of 2020, presented in Figure 15. It can be seen, particularly from the standard deviation indicated in the legend of Figure 15 (more reliable to assess the GIMs with altimeter VTEC measurements, see [24]), that slightly better performances are still obtained in the context of experimental IGS RT-GIMs (2.78 and 2.80 TECUs for UPC-IonSAT and CAS one, in front of 2.98 TECUs for CNES).

## 5. Conclusions

This research aimed to assess the possibility of using real-time SSR ionospheric corrections from CNES provided via RTCM streams to perform medium-range (up to 82 km) instantaneous RTK measurements. For this purpose, the authors performed computational tests both in real time and postprocessing. Test data consisted of 3300 epochs of GPS measurements collected from a receiver mounted on the roof of a car moving in urban conditions. The results were compared with a reference trajectory obtained from processing a short baseline. In the case of the 82 km baseline in real-time solution, 16% of epochs were correctly solved without the use of SSR corrections, and nearly 56% of epochs were solved with the use of the SSR corrections, respectively. The percentage of wrong fixes dropped from 16% to 5% when SRR corrections were applied. The postprocessed solution achieved 1% and 30% correct ambiguity fixes without and with the use of the ionospheric corrections, respectively. At the same time, the ambiguity validation failure rate dropped from 85% and 8% in this variant. Nevertheless, both solutions using SSR (CNES CLK90, RTCM 1264) ionospheric corrections or UQRG IONEX resulted in solving 3.4 times more epochs in real time, and over 44 times more in postprocessing. What is even more important is that the improvement in both solutions is similar in reliability. Additionally, the obtained residual values are similar to those obtained in other studies performed in postprocessing and presented in the literature [17]. A multi-baseline solution calculated in postprocessing, as it might be expected, provided the best results, correctly solving 72% of epochs with only 0.11% of wrong fixes. Therefore, our further studies will aim at the implementation of this mode into real-time software.

Our initial results indicate that the use of corrections calculated on the basis of spherical harmonics provided in the RTCM 1264 message significantly increases the success rate and reliability of the instantaneous precise positioning. A further improvement in the results is expected when the International GNSS Service starts its operational real-time ionospheric product. In particular, it has been shown that other experimental IGS RT-GIMs, from CAS and UPC-IonSAT, have recently behaved similarly or slightly better than CNES, opening the way for a potentially better IGS-combined RT-GIM. In addition, real-time multi-station solutions shall clearly increase the success rate of the ambiguity fixing, bringing closer the operational application of the instantaneous RTK over longer baselines.

## Figures and Tables

**Table 1:** RTCM streams recorded during the test drive.

Source	RTCM Data
Topcon NET-G5	1004, GPS raw measurement
BROD (82 km) reference station	1004, GPS raw measurement 1006, station coordinates
KRO1 (1–6 km) reference station	1004, GPS raw measurement 1006, station coordinates
CLK90	1264, SSR VTEC RTCM message
IGC01	1058, SSR SV eph and clock corrections
RTCM3EPH	1019, GPS satellites ephemeries

**Table 2:** pyGNSS real-time processing parameters.

Parameter	Value
GNSS system and signals	GPS L1 and L2
Satellite orbits and clocks	Broadcast + SSR correction
Positioning model	relative, ionosphere and troposphere weighted
First data epoch	9:35 GPST
Last data epoch	10:30 GPST
Data interval	1 second
Session length	3300 epochs
Ambiguity resolution	LAMBDA
Ambiguity selection validation	Ratio-test
Reference frame	ETRF2000
Min # of satellites	4
Min satellite elevation	15
Min satellite SNR	40

**Table 3:** Statistics of real-time solutions.

Ref. Station	Baseline (km)	ARSR (%)	AVFR (%)	ARSR_True (%)	Iono Corr.
KRO1	1–6 km	86.22	-	-	no
BROD	81.7	19.34	15.68	16.31	no
BROD	81.7	58.66	5.03	55.71	yes

**Table 4:** Processing parameters.

Parameter	Value
GNSS system and signals	GPS L1 and L2
Satellite orbits and clocks	Int. GNSS Service (IGS) ultrarapid (predicted part)
Positioning model	relative, ionosphere and troposphere weighted
First data epoch	9:35 GPST
Last data epoch	10:30 GPST
Data interval	1 second
Session length	3300 epochs
Ambiguity resolution	LAMBDA
Ambiguity selection validation	W-test
Reference frame	ETRF2000
PDOP threshold	7
W-test threshold	3
Min # of satellites	4

**Table 5:** Statistical summary of all postprocessed solutions.

Ref. Station	Baseline [km]	ARS(%)	AVFR (%)	ARSR_True (%)	Iono. Corr.
KRO1	1–6 km	77.48	-	-	no
DZIA	61.8	35.88	0.87	35.56	no
DZIA	61.8	47.98	0.41	47.78	yes
BROD	81.7	4.61	85.21	0.68	no
BROD	81.7	33.09	8.04	30.43	yes
MULTI3	60.6–81.7	61.67	0.13	61.59	no
MULTI3	60.6–81.7	71.60	0.11	71.52	yes
